# A case of needle tract seeding that seemed to be caused by endoscopic ultrasound‐guided fine‐needle aspiration

**DOI:** 10.1002/ccr3.7043

**Published:** 2023-03-07

**Authors:** Masanari Sekine, Takeharu Asano, Risako Kurabayashi, Shimpei Maeda, Fumiaki Watanabe, Hiroshi Noda, Toshiki Rikiyama, Hirosato Mashima

**Affiliations:** ^1^ Department of Gastroenterology, Saitama Medical Center Jichi Medical University Saitama Japan; ^2^ Department of Surgery, Saitama Medical Center Jichi Medical University Saitama Japan

**Keywords:** endoscopic ultrasound‐guided fine‐needle aspiration, needle tract seeding, pancreatic ductal adenocarcinoma

## Abstract

A 66‐year‐old man underwent a single endoscopic ultrasound‐guided fine‐needle aspiration (EUS‐FNA) session and distal pancreatectomy for the pancreatic body adenocarcinoma measuring 12 mm in diameter. At 3 years after surgery, we diagnosed needle tract seeding (NTS) and performed total gastrectomy. NTS can occur with small tumors or after a single session of EUS‐FNA.

## Graphical abstract

It seems difficult to avoid NTS after EUS‐FNA. We need careful follow‐up for patients who underwent EUS‐FNA for tumors located in the pancreatic body and tail. In addition to CT and/or PET‐CT, EGD is useful for follow‐up.

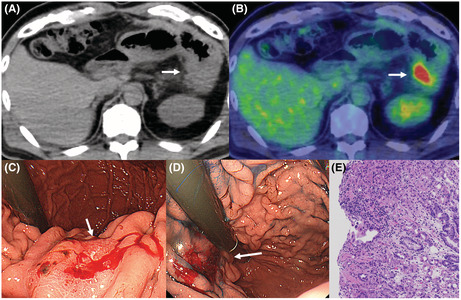

## 1 INTRODUCTION

Endoscopic ultrasound‐guided fine‐needle aspiration (EUS‐FNA) is widely used as a first‐line procedure for the diagnosis of pancreatic solid tumors. However, EUS‐FNA is known to be associated with some serious adverse events. The rate of early adverse events, including acute pancreatitis, bleeding, infection, and duodenal perforation, is reported to be 0.98%–3.4%.^1^, ^2^, ^3^, ^4^, ^5^ Recently, the incidence rate of needle tract seeding (NTS), one of the late adverse events, after EUS‐FNA, is reported to be 0.33% for all primary pancreatic tumors.^6^ We experienced a case involving a gastric tumor that seemed to be derived from NTS in a patient who underwent distal pancreatectomy after being diagnosed with pancreatic ductal adenocarcinoma (PDAC) based on EUS‐FNA. In this case, the samples collected in a single session of EUS‐FNA were sufficient to make a diagnosis. We report the details of this case and related cases described in the relevant literature.

## 2 CASE REPORT

**FIGURE 1:**
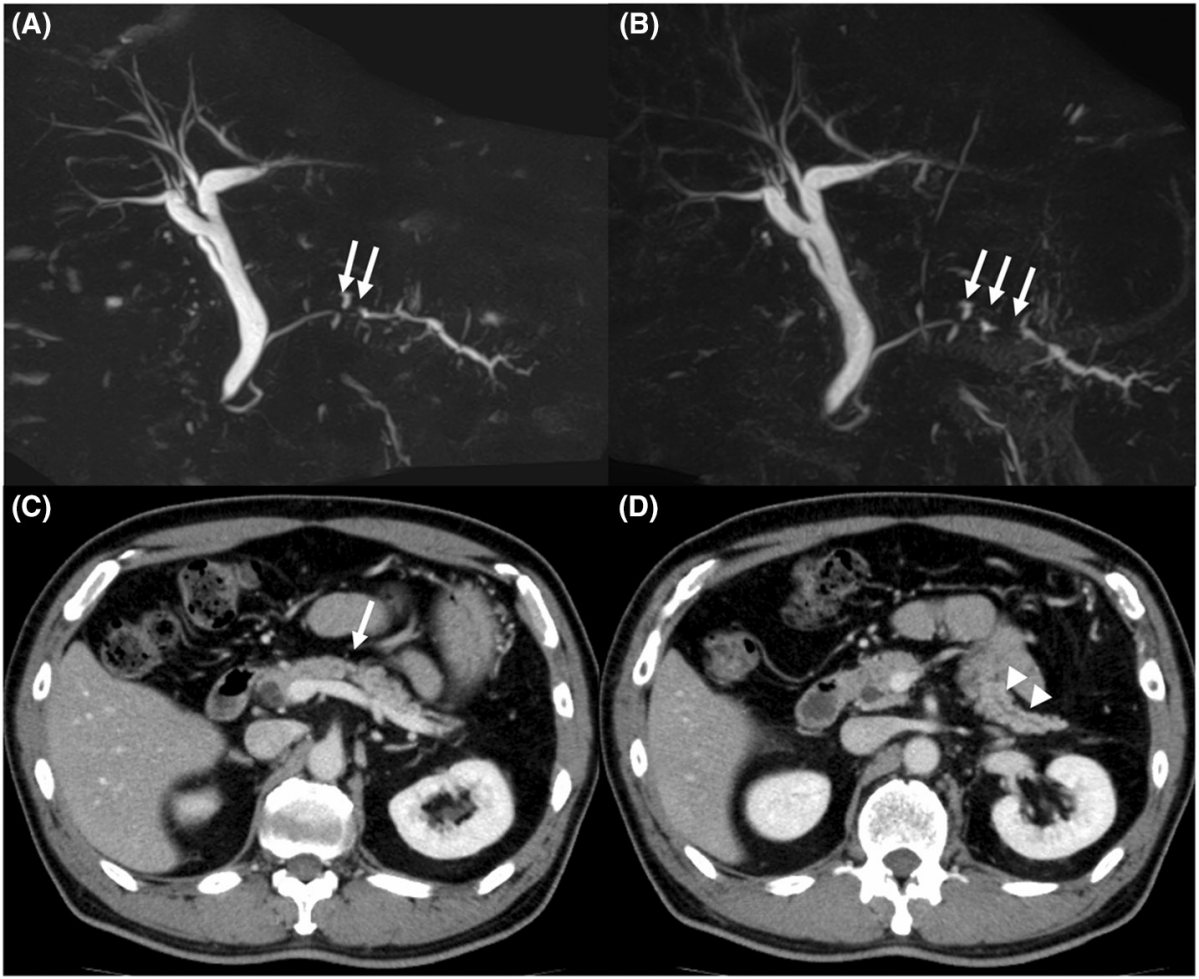
MRCP findings and CT findings. (A): MRCP showed stenosis of the MPD (arrow) in referral to our hospital. (B): MRCP showed that the stenosis of the MPD (arrow) became longer after 7 months. (C): CT showed that the pancreatic parenchyma of the body was atrophic (arrow). (D): CT showed that MPD of the tail was mildly dilated (arrow head).

**FIGURE 2:**
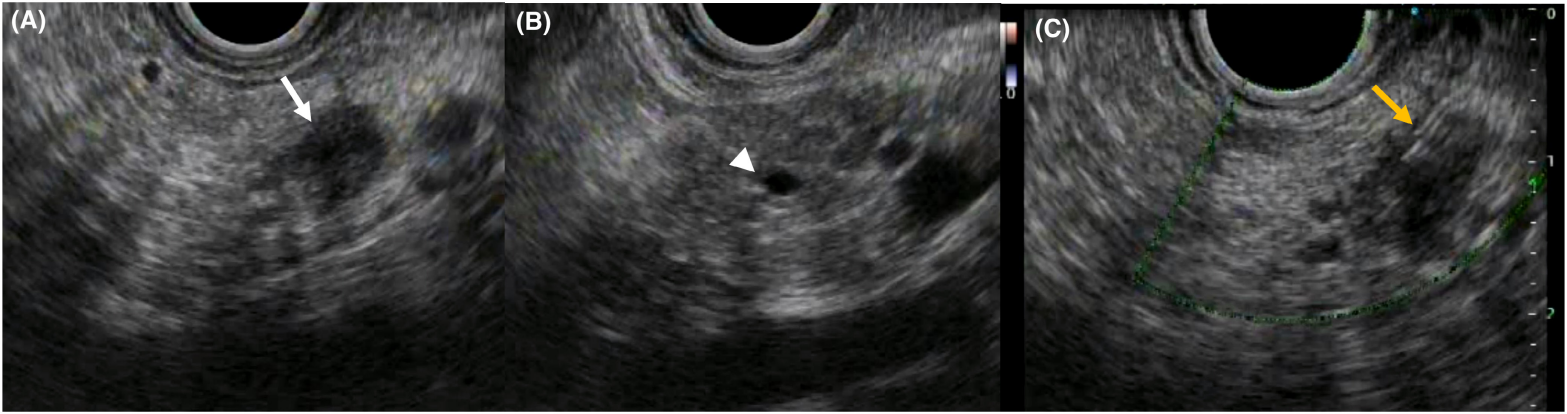
EUS findings. (A): EUS showed a low echoic tumor of 7 mm in diameter in the pancreatic body (white arrow). (B): The MPD in the pancreatic tail was dilated (arrow head). (C): EUS‐FNA was performed for the pancreatic body tumor using a 22G lancet needle (yellow arrow).

**FIGURE 3:**
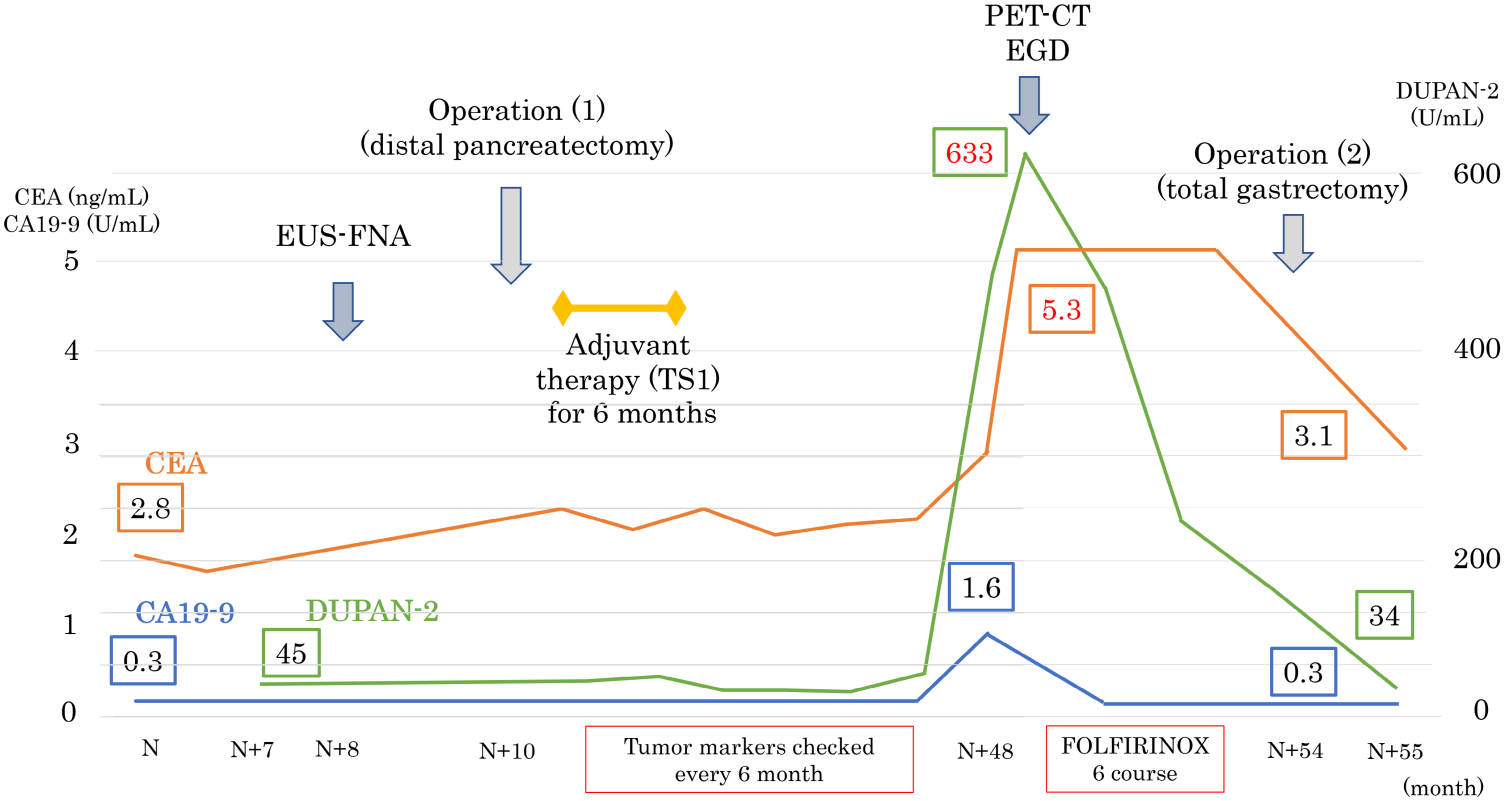
The transition of tumor markers and the clinical course. CA19‐9 level was not elevated, but CEA and DUPAN‐2 levels were elevated at 3 years after distal pancreatectomy. CEA and DUPAN‐2 levels fell to the normal range after total gastrectomy.

**FIGURE 4:**
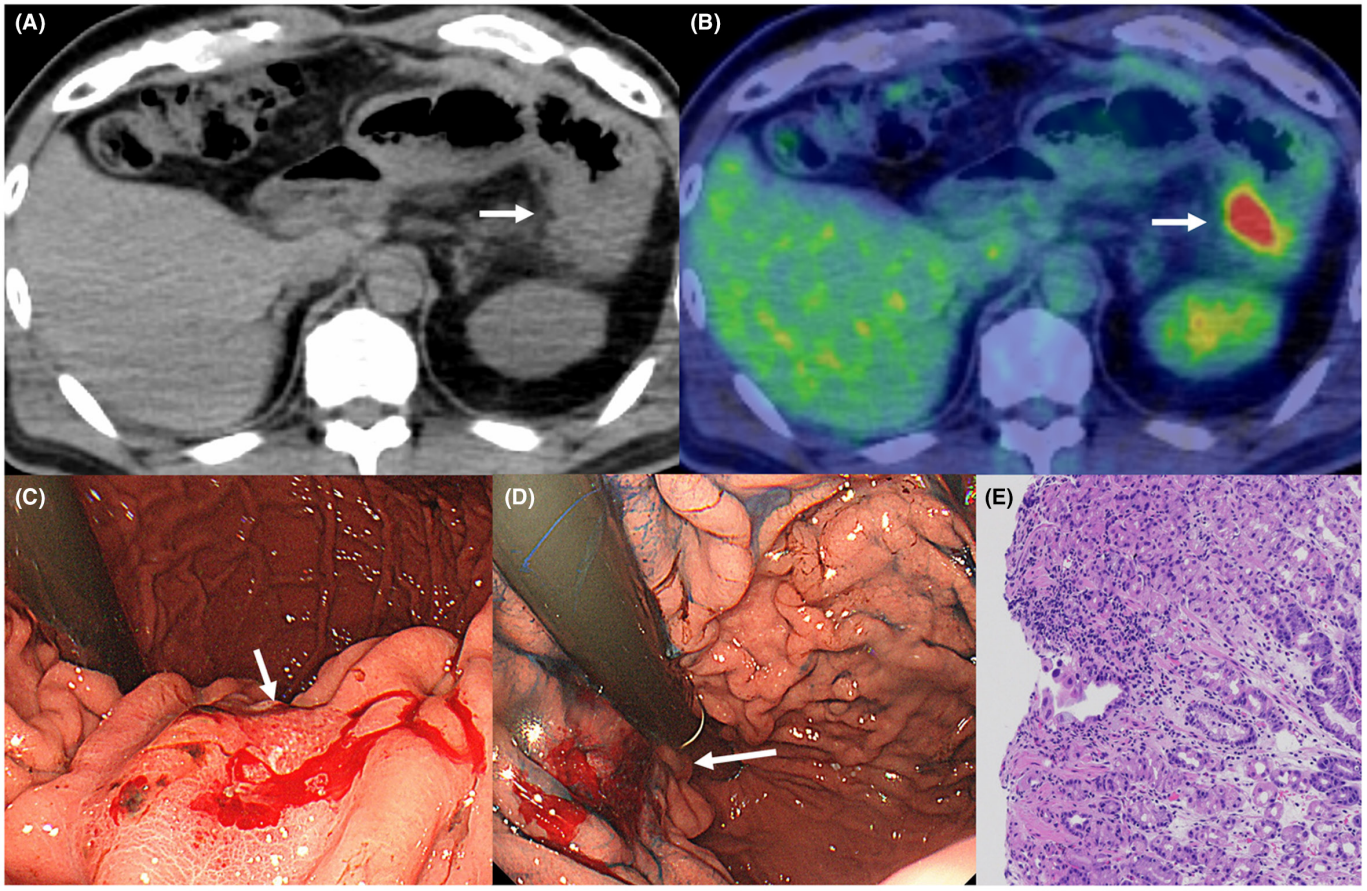
PET‐CT findings and EGD findings. (A, B): Plain CT and PET‐CT showed a subepithelial lesion‐like gastric lesion (arrow). (C, D): EGD showed that the tumor was 20 mm in size, reddish, and had a central depression (arrow). It was located on the posterior wall of the upper body of the stomach (arrow). (E): The pathological findings of the biopsy specimen indicated adenocarcinoma.

**FIGURE 5:**
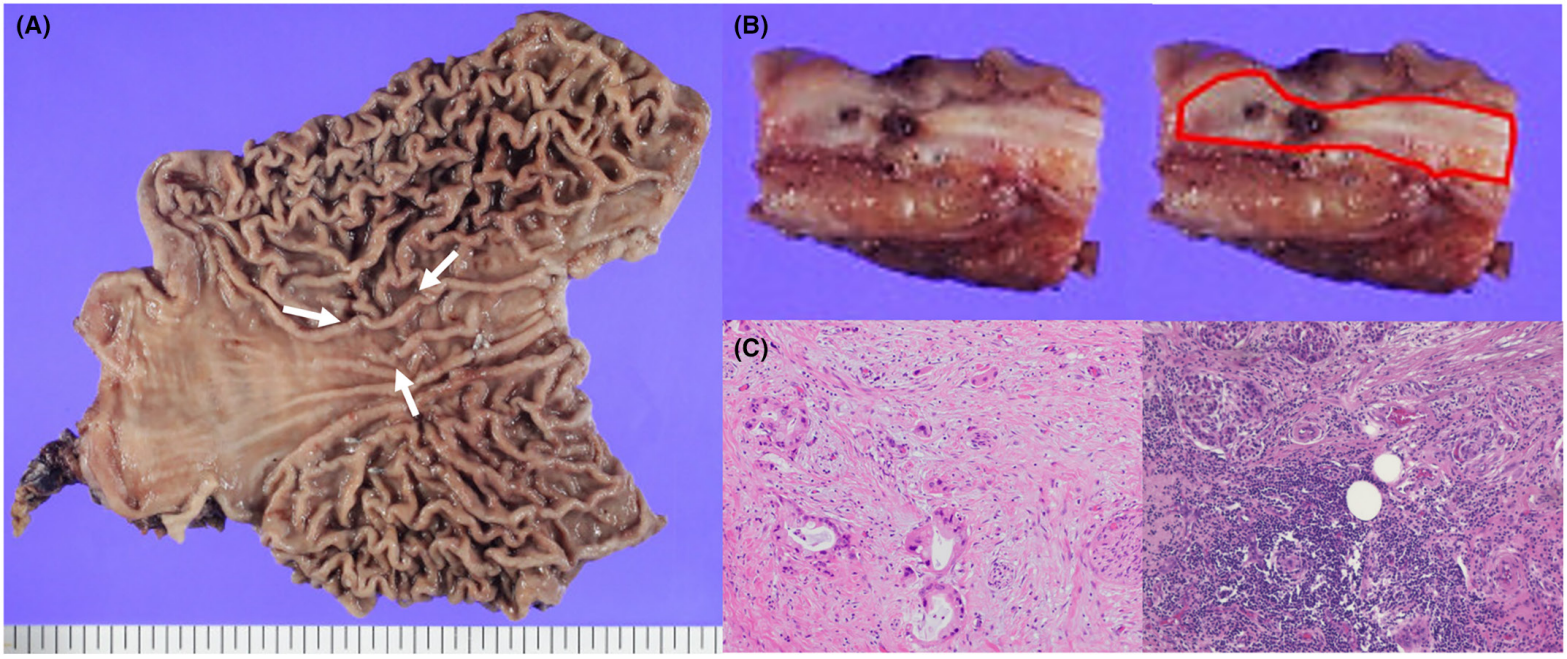
The pathological findings after total gastrectomy. (A): Examination of the surgically resected specimen revealed a depressed lesion (arrows). (B): Macroscopic observation of the resected gastric tumor revealed a white hard tumor (red line). (C): The pathological findings of hematoxylin and eosin staining indicated adenocarcinoma (left side), similar to the originally resected pancreatic body adenocarcinoma (right side).

The patient was a 66‐year‐old man who was referred to our hospital for main pancreatic duct (MPD) stenosis that was detected on magnetic resonance cholangiopancreatography (MRCP) (Figure 1A). No tumor was detected on EUS; thus, he was followed as an outpatient. After 7 months, the stenosis of the MPD became longer on MRCP (Figure 1B). Computed tomography (CT) showed that the pancreatic parenchyma of the pancreatic body was atrophic (Figure 1C), and the MPD of the pancreatic tail was mildly dilated (Figure 1D). EUS showed a low echoic tumor of 7 mm in diameter in the pancreatic body (Figure 2A). The MPD was not dilated from the head to the tumor but was dilated in the tail (Figure 2B). We performed EUS‐FNA with a 22G lancet needle (Figure 2C). Sufficient specimens were obtained in a single session and rapid on‐site evaluation (ROSE) suggested adenocarcinoma. The preoperative diagnosis was pancreatic adenocarcinoma (cStageIA). Distal pancreatectomy was performed without preoperative chemotherapy. The final diagnosis was pancreatic adenocarcinoma (pStageIIA, 12 mm, pT3, INFb, ly1, v1, ne0, S0, RP1, PV0, A0, PL0, OO0, PCM0, DPM0, pN0). After surgery, S‐1 was orally administered for 6 months as adjuvant therapy, and then he was followed up every 3–6 months. The patient's CA19‐9 level was not elevated; however, his CEA and DUPAN‐2 levels were elevated at 3 years after surgery (Figure 3). Positron emission tomography‐computed tomography (PET‐CT) showed a sub‐epithelial lesion‐like gastric lesion (Figure 4A,B); thus, esophagogastroduodenoscopy (EGD) was performed. The tumor was 20 mm in size, reddish, and had a central depression (Figure 4C,D). It was located on the posterior wall of the upper body of the stomach. We diagnosed NTS based on the past medical history of EUS‐FNA, tumor localization, and the histology of biopsy specimens (Figure 4E). However, we could not exclude the possibility of peritoneal dissemination and administered six courses of chemotherapy (FOLFILINOX) prior to surgery. The tumor was reduced in size and there was no apparent peritoneal dissemination. We then performed total gastrectomy (Figure 5A), because of the location of the tumor (upper body of the stomach) and the difficulty to determine the spread of the tumor.

The tumor was confined within the gastric wall (Figure 5B). The pathological findings indicated adenocarcinoma, similar to the originally resected pancreatic body adenocarcinoma (Figure 5C). The CEA and DUPAN‐2 levels decreased to the normal range after surgery (Figure 3).

## 3 DISCUSSION

EUS‐FNA is a common diagnostic procedure for pancreatic tumors, for which it has pooled sensitivity and specificity of 85%–92% and 96%–98%, respectively.^7^, ^8^ A low rate of adverse events has been reported.^1^, ^2^ However, recently, NTS has been reported after EUS‐FNA for all types of primary pancreatic tumors including PDAC.^6^

Kitano et al. reported that the incidence of NTS in PDAC was 0.409%.^6^ Most cases of NTS were detected after transgastric EUS‐FNA, and NTS occurred in the gastric wall. There were no cases of NTS after transduodenal EUS‐FNA. The reason is probably that the puncture route in the gastric wall remain after distal pancreatectomy, while the route in the duodenal wall is resected in pancreatoduodenectomy.

The difference between peritoneal dissemination and NTS has been debated. The diagnosis is related to whether the tumor originates from the puncture route.^9^ In this case, we consider that the tumor had arisen from the submucosal or muscular layer because the tumor was mainly confined within the gastric wall. The tumor was located on the posterior wall of the upper body of the stomach, compatible with the puncture route. Therefore, we diagnosed as NTS, not peritoneal dissemination, although we could not completely rule out the possibility of intramural metastasis.

The factors that cause NTS are still unknown. Sakamoto et al. reported that using a needle with a side‐hole and the slow‐pull technique might cause leakage of malignant specimens.^10^ EUS‐FNA for a large tumor and/or multiple sessions may be related to NTS.^9^, ^11^, ^12^, ^13^ A small number of sessions in EUS‐FNA may be preferable to reduce NTS.

The previous report showed that the median overall survival (OS) and recurrence‐free survival (RFS) in patients with or without EUS‐FNA did not differ to a statistically significant extent.^14^ In addition, when NTS occurred, the survival time was significantly longer in patients who underwent NTS resection than in those who did not.^6^

For a small pancreatic tumor including PanIN, endoscopic retrograde cholangiopancreatography (ERCP) is another common diagnostic procedure. The sensitivity and specificity of ERCP‐assisted pancreatic juice cytology including serial pancreatic juice aspiration cytologic examination (SPACE) was 54.9%–100% and 83%, respectively.^15^, ^16^ One of the major adverse events is post‐ERCP pancreatitis (PEP) and the incidence of PEP was reported to be 2.6%–9.7%.^17^, ^18^, ^19^, ^20^

In the diagnosis of pancreatic tumors located in the pancreatic body and tail, we need to select EUS‐FNA and/or ERCP, or performed upfront surgery without a pathological diagnosis.

Recently, neoadjuvant chemotherapy has become common because neoadjuvant chemotherapy using gemcitabine and S‐1 for PDAC has demonstrated a survival benefit in comparison to upfront surgery (Prep‐02/JSAP05).^21^ Therefore, it is becoming more important to make a preoperative pathological diagnosis. Due to the high accuracy rate and the low adverse event rate, EUS‐FNA may be preferred over ERCP for making a preoperative pathological diagnosis when a tumor is detected on EUS.

In the therapy of NTS, surgery is recommended. Kitano et al.^6^ reported the overall survival time was significantly longer in patients who underwent NTS resection (median 51.9 vs. 26.2 months, *p* = 0.037). Detailed surgical records were not shown in their report, and we searched the previous reports in which the way of surgery was described (Table 1).^10^, ^12^, ^22^, ^23^, ^24^, ^25^, ^26^, ^27^, ^28^, ^29^, ^30^, ^31^, ^32^ Partial gastrectomy was selected frequently (64.3%), then, total gastrectomy (21.4%) and partial gastrectomy (14.3%). Regarding detection devices, NTS lesions were detected in 42.9% by EGD, 42.9% by CT, and 14.3% by PET‐CT.

**TABLE 1:** Reported case of gastrectomy for needle tract seeding after EUS‐FNA of a pancreatic carcinoma.

Author	Year	Age	Pancreatic primary tumor					Recurrence tumor				
			Location	Tumor size (mm)	Primary treatment	pStage	Pathological feature	Time interval (months)	Detection device	Finding of EGD	Surgical type	Pathological feature
Ahmed^22^	2011	75	Pb	Unknown	MSPP	pT2N0M0	adenocarcinoma	39	PET‐CT	SEL	TG	adenocarcinoma
Minaga^23^	2015	64	Pb	26	DP	pT3N0M0	moderately differentiated tubular adenocarcinoma	8	EGD	SEL	PG	moderately differentiated tubular adenocarcinoma
Sakurada^24^	2015	87	Pb	25	DP	pT3N0M0	adenosquamous carcinoma	19	CT	SEL	PG	squamous cell carcinoma
Tomonari^25^	2015	78	Pb	25	DP	pT3N0M0	adenocarcinoma	9	EGD	SEL	TG	well differentiated adenocarcinoma
Minaga^26^	2016	72	Pb	10	DP	pT1N0M0	adenocarcinoma	10	EGD	SEL with depression	PG	adenocarcinoma
Iida^27^	2016	78	Unknown	Unknown	DP	pT3N0M0	adenocarcinoma	6	EGD	SEL	DG	adenocarcinoma
Sakamoto^10^	2018	50	Pt	38	DP	pT4N1M0	adenocarcinoma	24	EGD	SEL with depression	PG	adenocarcinoma
Kawabata^28^	2019	78	Pb	46	DP	pT2N0M0	moderately differentiated tubular adenocarcinoma	24	CT	SEL	PG	moderately differentiated tubular adenocarcinoma
Hayasaka^29^	2020	75	Pt	Unknown	DP	pT1N0M0	adenocarcinoma	8	CT	SEL	PG	adenocarcinoma
Sato^12^	2020	83	Pb	32	DP	pT3N1M0	adenocarcinoma	25	CT	SEL	PG	adenocarcinoma
Rothermel^30^	2020	61	Pb	37	DP	pT3N0M0	well‐differentiated tubular adenocarcinoma	42	EGD	SEL with depression	PG	adenocarcinoma
Nagano^31^	2021	67	Pb	20	DP	pT3N0M0	adenocarcinoma	34	CT	SEL	DG	adenocarcinoma
Ogura^32^	2021	80 s	Pt	Unknown	DP	pT3N0M0	adenocarcinoma	12	CT	SEL	PG	adenocarcinoma
Our case	2022	66	Pb	12	DP	pT3N0M0	well‐differentiated tubular adenocarcinoma	36	PET‐CT	SEL with depression	TG	adenocarcinoma

Abbreviations: CT, computed tomography; DG, distal gastrectomy; DP, distal pancreatectomy; EGD, esophagogastroduodenoscopy; MSPP, middle‐segmental‐preserving pancreatectomy; Pb, pancreatic body; PET‐CT, positron emission tomography‐computed tomography; PG, partial gastrectomy; Pt, pancreatic tail; SEL, subepithelial lesion; TG, total gastrectomy.

In the present case, the tumor was small but was detectable on EUS, and a single session of EUS‐FNA could collect sufficient specimens to make the diagnosis. However, NTS occurred. Currently, it seems difficult to avoid NTS. Thus, caution is required when performing EUS‐FNA for tumors located in the pancreatic body and tail. Even in cases in which EUS‐FNA is performed only once, we may need to perform EGD with particular attention paid to the posterior wall of the stomach during follow‐up in addition to CT and/or PET‐CT. When NTS occurs, the NTS lesion should be resected.

## AUTHOR CONTRIBUTIONS

Masanari Sekine: Have made substantial contributions to conception and design, or acquisition of data, or analysis and interpretation of data and been involved in drafting the manuscript or revising it critically for important intellectual content. Takeharu Asano: Been involved in drafting the manuscript or revising it critically for important intellectual content. Risako Kurabayashi: Have made substantial contributions to conception and design, or acquisition of data, or analysis and interpretation of data. Shimpei Maeda: Have made substantial contributions to conception and design, or acquisition of data, or analysis and interpretation of data. Fumiaki Watanabe: Have made substantial contributions to conception and design, or acquisition of data, or analysis and interpretation of data. Hiroshi Noda: Been involved in drafting the manuscript or revising it critically for important intellectual content. Toshiki Rikiyama: Been involved in drafting the manuscript or revising it critically for important intellectual content. Hirosato Mashima: Given final approval of the version to be published. Each author should have participated sufficiently in the work to take public responsibility for appropriate portions of the content and Agreed to be accountable for all aspects of the work in ensuring that questions related to the accuracy or integrity of any part of the work are appropriately investigated and resolved.

## ACKNOWLEDGMENTS

None.

## CONFLICT OF INTEREST STATEMENT

The authors have no conflict of interest to declare.

## INFORMED CONSENT

Written informed consent was obtained from the patient to publish this report in accordance with the journal's patient consent policy.

## Data Availability

Data sharing not applicable to this article as no datasets were generated or analysed during this study.
